# Surveillance of *tuberculosis* (TB) cases attributable to relapse or reinfection in London, 2002-2015

**DOI:** 10.1371/journal.pone.0211972

**Published:** 2019-02-15

**Authors:** Baharak Afshar, Jacqueline Carless, Anita Roche, Sooria Balasegaram, Charlotte Anderson

**Affiliations:** 1 Field Service, National Infection Service, Public Health England, London, United Kingdom; 2 European Public Health Microbiology Programme (EUPHEM), European Centre for Disease Prevention and Control (ECDC), Stockholm, Sweden; University of Liverpool, UNITED KINGDOM

## Abstract

Recurrence of TB in an individual can occur due to relapse of the same strain or reinfection by a different strain. The contribution of reinfection and relapse to TB incidence, and the factors associated with each are unknown. We aimed to quantify and describe cases attributable to relapse or reinfection, and identify associated risk factors in order to reduce recurrence. We categorised recurrent TB cases from notifications in London (2002–2015) as relapse or reinfection using molecular (MIRU VNTR strain type) and epidemiological information (hierarchical approach using time since notification, site of disease and method of case finding). Factors associated with each outcome were determined using logistic regression in Stata Version 13.1 (2009–2015 only). Of 43,465 TB cases, 1.4% (618) were classified as relapse and 3.8% (1,637) as reinfection. The proportion with relapse decreased from 2002 (2.3%) to 2015 (1.3%), while the proportion of reinfection remained around 4%. Relapse was more common among recent migrants (<1 year, odds ratio (OR) = 1.99, *p* = 0.005), those with a social risk factor (OR = 1.51, *p* = 0.033) and those with central nervous system, spinal, miliary or disseminated TB (OR = 1.75, *p* = 0.001). Reinfection was more common among long term migrants (>11 years, OR = 1.67, *p* = <0.001), those with a social risk factor (OR = 1.96, *p* = <0.001) and within specific areas in London. Patients with social risk factors were at increased risk of both relapse and reinfection. Characterising those with relapsed disease highlights patients at risk and factors associated with reinfection suggest groups where transmission is occurring. This will inform TB control programs to target appropriate treatment and interventions in order to reduce the risk of recurrence.

## Introduction

Recurrence of TB in an individual can occur due to a regrowth of the same strain of *Mycobacterium tuberculosis* that caused the previous TB episode, known as relapse, or reinfection by a different strain [1]. A host of factors can influence the likelihood of recurrent tuberculosis including the level of adherence to treatment, the severity of the original episode, the patient's immune status and the risk of reinfection [2].

In a low incidence setting, the risk of reinfection and subsequent disease is generally considered to be small and the majority of cases of recurrent tuberculosis would be expected to be due to reactivation (caused by relapse of a previously treated TB episode). In contrast, in high incidence settings the proportion of recurrent tuberculosis cases due to reinfection is higher because of the increased risk of exposure, especially in the presence of high prevalence of coexisting human immunodeficiency virus (HIV) [3]. Studies carried out in countries of medium incidence suggest that recurrence is more commonly caused by relapse, although the rate of reinfection could still play an important role [1,2].

Rates of tuberculosis (TB) in London have started to decrease in recent years, but it still has the highest burden in Western Europe [4]. The relative contribution of recurrent TB on the overall annual TB incidence and the influence of relapse or reinfection is likely to vary depending on epidemiological features of the endemic areas within London.

The National TB typing service in England was established in 2010 and since then all TB isolates have been typed using 24 loci mycobacterial interspersed repetitive units—variable number of tandem repeats (MIRU-VNTR), although since January 2018 whole genome sequence typing has replaced MIRU-VNTR in London. The definition of relapse or exogenous reinfection is based on the genotypic profile of the strains responsible for each TB episode. An indistinguishable MIRU-VNTR pattern, or one with 1 variation at MIRU-VNTR unit is considered consistent with relapse, while a different profile is suggestive of exogenous reinfection [5].

TB remains a serious public health problem in London. The relative contribution of TB reinfection and relapse to the overall incidence and the risk factors associated with each type of recurrent TB are not well-known. In 2015, 2,269 new cases of tuberculosis (TB) were notified among London residents, a rate of 26 per 100,000 population [6]. This was a 12% decrease from the rate observed in 2014, and a 38% decrease from 2011. Compared to the rest of the UK, however, rates remained highest in London and some areas within the city have a very high burden of disease (average rates between 2014 and 2016 over 40 per 100,000 in 5 local boroughs). The majority of cases occur among individuals born outside the UK, but rates have decreased in this population since 2011 [4]. The rate of TB in the UK born London population also decreased, but remains more than double in England overall.

Only a small proportion (6%) had a self-reported previous history of TB. Whether this is due to relapse or reinfection has not been studied. Currently we have limited information on proportion of each type of recurrent TB cases in London, their relative contribution to TB incidence and the characteristics that are associated with either relapse or reinfection. Information about the epidemiological and microbiological characteristics of recurrent TB is an important issue for public health programs as it highlights risks associated with history of TB relapse amongst individuals, identifies areas where ongoing transmission is occurring (so targeted interventions such as active case finding can be done) and it would ensure that appropriate health control strategies are followed for current patients to prevent further episodes of illness. Recurrence rates can also be used to assess the effectiveness of TB control programs that are in place.

The main objectives of this study were to determine the number and proportion of TB cases (in London) attributable to relapse or reinfection based on surveillance data from 2002 to 2015 and to identify and describe characteristics associated with either relapse or reinfection (using data from 2009 to 2015).

## Material and methods

### Study design and population

This study was a cross-sectional study conducted in London, an urban area of 1,738 square Km, whose census population was 10,236,000 inhabitants in 2015. The cohort of 44,000 TB patients was identified using the London TB Register of cases notified via the routine Public Health England tuberculosis reporting system from 1 January 2002 through 31 December 2015 [6,7]. Strain typing on patients was only obtainable from 2010 and information on social risk factors was available from 2009.

### Case definition

**Fig 1:**
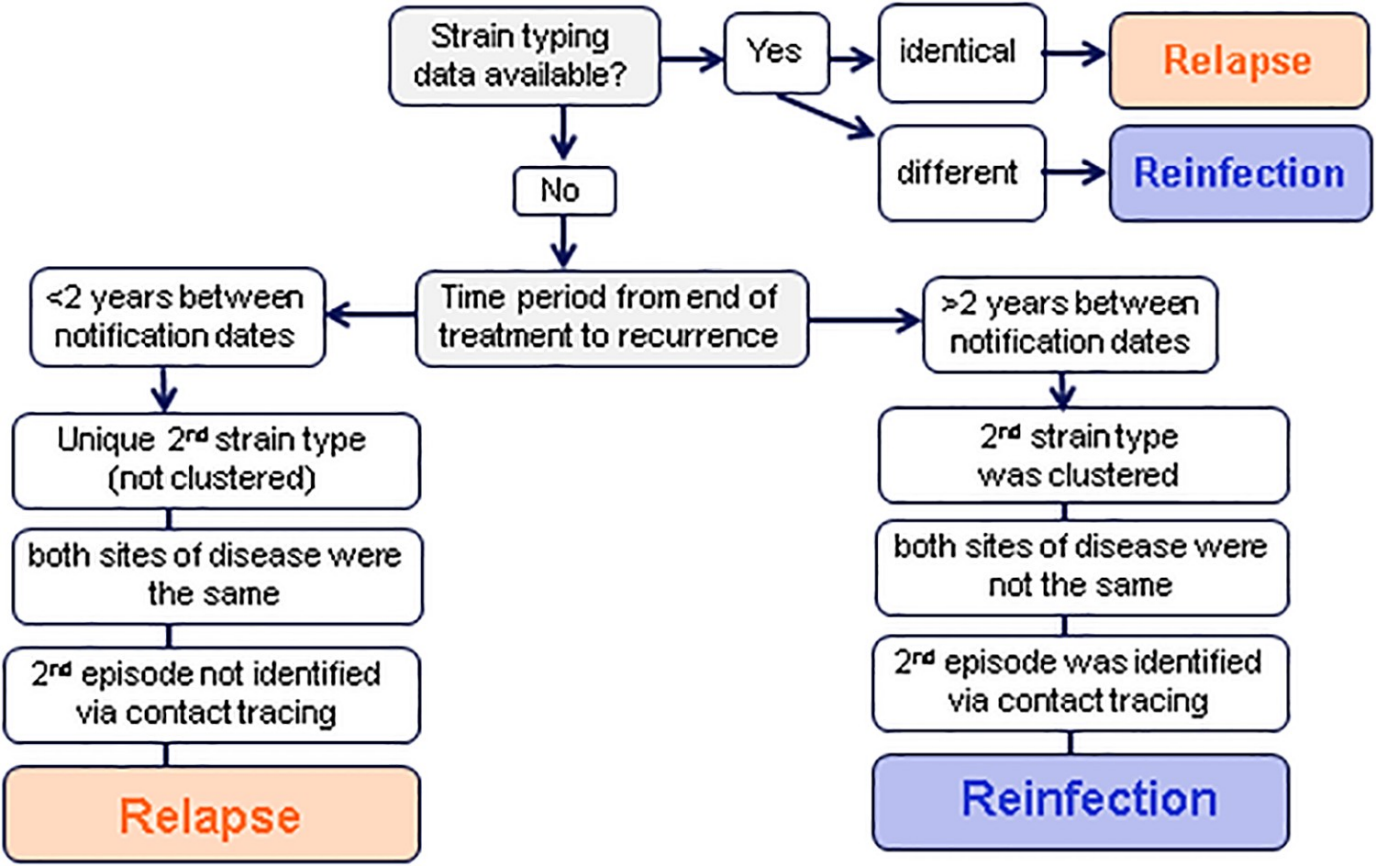
Categorising recurrent TB cases into relapse and reinfection.

An episode of recurrent tuberculosis was defined as a patient who reported a previous diagnosis of TB. For patients with a previous history of tuberculosis treated in London, those who did not complete their previous course of treatment were excluded as their illness may otherwise be an extension of their original episode. Patients without information on their past treatment were assumed to have completed a standard course of therapy. Recurrence was assigned to either:

Relapse: due to the same TB infection as caused the original TB illness or reinfection: due to infection with a different strain following successful TB treatment (Fig 1). Patients with a previous history of tuberculosis were categorised as relapse or reinfection according to microbiological, clinical and epidemiological information in a hierarchical method. If strain typing was available for both episodes: cases were defined as relapse if the strain types were indistinguishable or 1 MIRU VNTR unit difference and reinfection if strain types were different. As strain typing was not available for both episodes for many patients, molecular (clustering) information on the latest isolate and epidemiological information was used as a proxy to determine the likelihood of relapse or reinfection. Time since previous TB illness was the most important factor, as the risk of relapse was known to be highest immediately following treatment [1]. As the end of any previous treatment was not known, time between previous and second notification of disease was used. If the second strain type was unique, the patient was less likely to have recently acquired the infection in the UK (therefore more likely to be relapse), whereas if the second strain type was part of a cluster, this could indicate recent (re)infection. Cases were classified as relapse if:

1) Less than two years between notification dates AND all three of the following factors:
  1. second strain type was unique (not clustered)
  2. both sites of disease were the same (pulmonary vs. extra-pulmonary)
  3. second episode was not identified via contact tracing

If strain typing was not available for both episodes, recurrent cases were classified as reinfection if:

2) Two or more years between notification dates AND all three of the following factors:
  1. second strain type was clustered
  2. both sites of disease were not the same (pulmonary vs. extra-pulmonary)
  3. second episode was identified via contact tracing

The remaining unclassified recurrent cases were then assigned to a cohort based on time between each notification plus two of the above factors. Those still unclassified were then assigned based on time between notification plus one factor. Finally, those remaining were classified based on time since previous notification. Recurrent cases with no information on time since previous notification were excluded from the analysis. Social risk factors were defined as current or history of homelessness, imprisonment, drug misuse or alcohol misuse.

### Data sources and collection

Data on TB cases in London came from the Public Health England (PHE) London TB Register (LTBR). The data contributes to the national Enhanced TB Surveillance (ETS) system [6,7]. Data collected includes notification details, demographic, clinical and microbiological information, including drug resistance and strain type, provided by the National Mycobacterium Reference Laboratory (NMRL). For the descriptive analysis we included all previously diagnosed TB cases from 2002 to 2015. For the multivariable analysis we only analysed adult TB cases from 2009 to 2015, as information on social risk factors was not collected prior to 2009.

### Data management

Data management and analysis were performed using Stata Version 13.1 and MS Excel. Data were de-duplicated, cleaned and stored in a bespoke database. All patient data were anonymized and stored on secure drives according to Caldicott principles and in compliance with the Data Protection Act (1998). All de-notified entries were also excluded from the analysis.

### Variables

The following variables were included in this study, and were collected as part of routine surveillance on all patients notified with tuberculosis: Socio-demographic variables: age, sex, ethnicity, country of origin, world region of birth, health protection team area and local authority of residence as well as time since UK entry (for those born abroad). Social risk factors: past or current prison history, homelessness, alcohol misuse and drug misuse. Clinical variables: TB recurrence, site of TB disease (pulmonary or/and extra-pulmonary forms including central nervous system, spinal, miliary or disseminated TB), history of TB treatment and *in vitro* susceptibility data of *M*. *tuberculosis* isolates (isoniazid and multidrug resistance (MDR); lack of susceptibility to both isoniazid and rifampicin), and MIRU VNTR strain typing data. For previously diagnosed patients, this information was taken from the latest episode only.

### Statistical analysis

Relapse and recurrent cases were defined according to the outcome definition. Cases were described according to the variables included. To investigate any associations between patient characteristics and being a case of relapse (vs. all other TB cases, including those due to reinfection) or reinfection (vs. all other TB cases, including relapse), univariable analysis and logistic regression were used. Exposure variables with a *p*-value (Wald test) of 0.05 or less in univariable analysis were included in a multivariable logistic regression model for each outcome along with age as a continuous variable. A backwards stepwise approach were used to identify a final model, eliminating variables with the highest *p*-values from likelihood ratio tests (*p* values >0.05) first and examining at each step for possible confounders. Multivariable analysis was presented as adjusted OR with 95% confidence intervals (CI). A two-tailed *p*-value of 0.05 or lower were considered statistically significant.

## Results

### Descriptive analysis of previously diagnosed TB cases

Of 44,000 cases of TB among London residents between 2002–2015, 2,790 (6.3%) reported a previous diagnosis of TB, (S1 Fig). The highest number of previously diagnosed TB cases was in 2009 (238/3,106; 7.7%) and since then has declined steadily to 142 cases in 2015 (of 2,240, 6.3%), although the proportion has remained between 6–7%.

Age and sex distribution of patients previously diagnosed with TB were similar to that for all TB cases. From 2005, a previous diagnosis was more common among non-UK born patients (7.3% compared to 5.6% among UK born patients). The most common countries of birth of patients reporting a previous diagnosis of TB were India (accounting for 23%), Somalia (15%) and Pakistan (8%). London TB patients of white ethnicity experienced the highest proportion of previously diagnosed TB in 2015 (9%). The proportion with a previous TB diagnosis also varied by area within London, from 4.7% to over 10% between 2002–2015. Where known, social risk factors were reported by 18% of patients with relapsed disease (49/276) and 17% of those with reinfection (134/794).

### Species and drug resistance

Of 2,790 previously diagnosed TB cases, less than half (1,256; 45%) were culture confirmed, of which 99% (1,247) were identified as *M*. *tuberculosis*. A higher proportion of those with pulmonary disease were culture confirmed (923/1,582; 58%) than those with exclusively extra-pulmonary TB (333/1,207; 28%). Among culture confirmed cases, 15% (190/1,240) of those with a previous diagnosis of TB were resistant to one or more first line drug compared to 9% of those without a previous diagnosis (1,881/20,980), with 5.9% (73) multi-drug resistant compared to 1.2% of those without a previous diagnosis (259).

### TB cases classified into relapse and reinfection

Of the 44,000 TB patients, 535 that reported previous TB were excluded from any further analysis as we were unable to classify them due to missing data (especially time since previous diagnosis). Of 2,255 remaining TB cases reporting previous TB, 618 (1.4%) were classified as relapse and 1,637 (3.8%) as reinfection. Only a small number were defined based on strain typing comparison of both episodes (4 defined as indistinguishable and therefore relapse, and 1 with 7 MIRU VNTR differences classed as reinfection). The majority of relapse cases met all of the definition of less than two years between notification dates, not in a cluster, and not found via contact tracing (545). Only 6 cases met all of the reinfection definition (more than two years between notification dates, new strain was in a cluster, and found via contact tracing), and a further 243 had more than two years between notification dates and either the new strain was in a cluster or they were found via contact tracing. The remainder with missing information on clustering and contact tracing were classified according to time between notification dates.

### Univariable analysis of relapse cases

**Fig 2:**
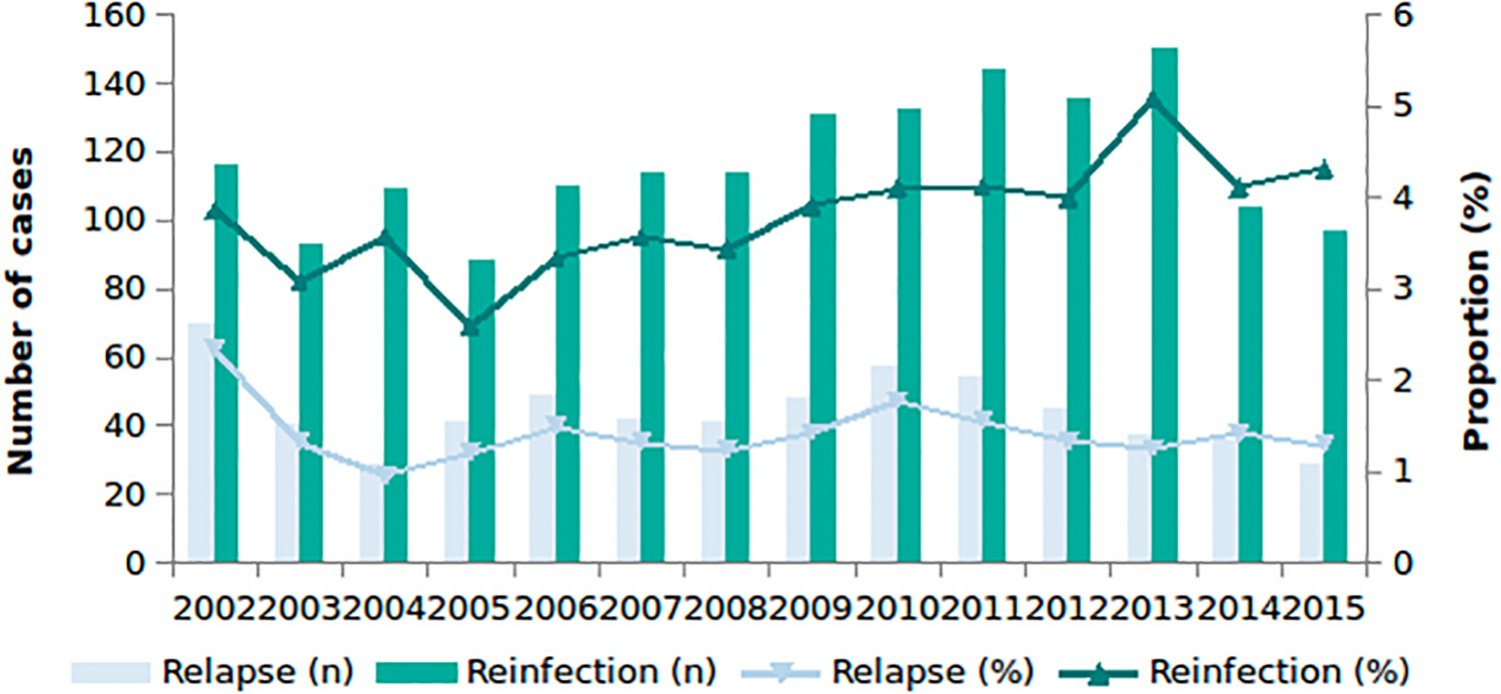
Number and proportion of relapse and reinfection cases from London by year, 2002 to 2015.

The proportion of cases due to relapse decreased from 2002 (2.3%) to 2015 (1.3%) (Fig 2). Relapse was more common among patients with social risk factors (homelessness, OR = 2.27, *p* = <0.001 and imprisonment, OR = 2.03, *p* = 0.01), followed by birth in Central Europe, OR = 2.07, *p* = 0.017 (vs. being born in Western Europe), recent entrants to the UK (<1 years since entry, OR = 1.90, *p* = 0.003; 2–5 years since entry, OR = 1.56, *p* = 0.033) (compared to being UK born) and those with central nervous system (CNS), spinal, miliary or disseminated disease (OR = 1.53, *p* = 0.007) (Table 1).

**Table 1:** Univariable analysis of relapse cases >16 years of age, London, 2009–2015.

	Exposure	Relapse TB cases			Non-relapse TB cases			Odds Ratio	95% confidence interval	p*
		Total	Exposed	%	Total	Exposed	%			
Sex	Male	298	164	55	19,802	11,637	58.8	0.86	[0.68–1.09]	0.194
Social risk factors	Any risk factors	256	38	14.8	17,539	1,826	10.4	1.5	[1.03–2.13]	0.021
	Homelessness	283	21	7.42	18,787	642	3.42	2.27	[1.37–3.57]	<0.001
	Prison history	278	14	5.04	18,737	478	2.55	2.03	[1.08–3.49]	0.01
	Drug use	277	16	5.78	18,697	690	3.69	1.6	[0.90–2.67]	0.069
	Alcohol use	258	14	5.43	17,731	782	4.41	1.24	[0.67–2.14]	0.431
Site of infection	CNS* TB	298	50	16.8	19,805	2,312	11.7	1.53	[1.10–2.08]	0.007
Time since entry into UK	UK born	271	33	12.2	17,415	2,770	15.9	Reference		
	0–1 years	271	58	21.4	17,415	2,562	14.7	1.9	[1.24–2.92]	0.003
	2–5 years	271	77	28.4	17,415	4,138	23.8	1.56	[1.04–2.36]	0.033
	6–10 years	271	42	15.5	17,415	2,864	16.5	1.23	[0.78–1.95]	0.375
	11+ years	271	61	22.5	17,415	5,081	29.2	1.01	[0.66–1.54]	0.972
Ethnic group	Indian	295	94	31.9	19,625	5,870	29.9	Reference		
	White	295	34	11.5	19,625	2,171	11.1	0.98	[0.66–1.45]	0.912
	Black Caribbean	295	4	1.36	19,625	655	3.34	0.38	[0.14–1.04]	0.06
	Black African	295	62	21	19,625	4,331	22.1	0.89	[0.65–1.23]	0.496
	Pakistani	295	25	8.47	19,625	1,963	10	0.8	[0.51–1.24]	0.312
	Bangladeshi	295	19	6.44	19,625	1,152	5.87	1.03	[0.63–1.69]	0.907
	Mixed/other	295	57	19.3	19,625	3,483	17.8	1.02	[0.73–1.42]	0.898
World region of birth	West Europe	283	39	13.8	19,054	3,231	17	Reference		
	Central Europe	283	15	5.3	19,054	599	3.14	2.07	[1.14–3.79]	0.017
	East Asia	283	3	1.06	19,054	198	1.04	1.26	[0.38–4.10]	0.706
	East Europe	283	4	1.41	19,054	158	0.83	2.1	[0.74–5.94]	0.163
	East Mediterranean	283	1	0.35	19,054	160	0.84	0.52	[0.07–3.79]	0.517
	North Africa	283	4	1.41	19,054	194	1.02	1.71	[0.60–4.83]	0.313
	North America & Oceania	283	1	0.35	19,054	41	0.22	2.02	[0.27–15.1]	0.492
	South Asia	283	142	50.2	19,054	8,975	47.1	1.31	[0.92–1.87]	0.137
	South East Asia	283	5	1.77	19,054	634	3.33	0.65	[0.26–1.66]	0.372
	South, Central America & the Caribbean	283	5	1.77	19,054	430	2.26	0.96	[0.38–2.46]	0.938
	Sub-Saharan Africa	283	64	22.6	19,054	4,291	22.5	1.24	[0.83–1.84]	0.301
	Unknown	283	0	0	19,054	143	0.75			
Age group	16–19	298	12	4.03	19,805	844	4.26	Reference		
	20–29	298	106	35.6	19,805	5,750	29	1.3	[0.71–2.37]	0.397
	30–39	298	92	30.9	19,805	5,195	26.2	1.25	[0.68–2.28]	0.478
	40–49	298	39	13.1	19,805	3,179	16.1	0.86	[0.45–1.66]	0.657
	50–59	298	23	7.72	19,805	2,062	10.4	0.78	[0.39–1.58]	0.5
	60–69	298	17	5.7	19,805	1,335	6.74	0.9	[0.43–1.88]	0.77
	70–79	298	8	2.68	19,805	983	4.96	0.57	[0.23–1.41]	0.22
	80+	298	1	0.34	19,805	457	2.31	0.15	[0.02–1.19]	0.07

*From Wald test.

### Multivariable analysis of relapse cases

Time since entry, age (linear and quadratic), site of disease (CNS, miliary, spinal or disseminated vs. any other) and having one or more social risk factor were included in the final model used to calculate adjusted Odds Ratios (aOR). Relapse was independently associated with being a recent migrant (<1 year since entry, aOR = 1.99, *p* = 0.005 compared to being UK born), having CNS, spinal, miliary or disseminated site of disease (aOR = 1.75, *p* = 0.001 compared to having any other site) and having a social risk factor (aOR = 1.51, *p* = 0.033) (Table 2).

**Table 2:** Multivariable analysis of risk factors for relapse, TB patients aged 16 years or older, London, 2009–2015.

Relapse	aOR**	[95% Conf. Interval]	*p*>|z|*
0–1 years since entry^1^	1.99	[1.23–3.23]	0.005
2–5 years since entry	1.62	[1.02–2.57]	0.039
6–10 years since entry	1.37	[0.83–2.28]	0.210
11+ years since entry	1.20	[0.74–1.94]	0.467
Age (continuous, linear)	0.99	[0.98–1.00]	0.0277
CNS TB^2^	1.76	[1.25–2.46]	0.0021
Any risk factors	1.53	[1.05–2.24]	0.0350

*Likelihood ratio test

**Adjusted Odds Ratio

^1^UK time of entry versus UK born

^2^CNS, spinal, miliary or disseminated disease

### Univariable analysis of reinfection cases

Of 43,465 TB cases from 2002–2015, 3.8% (1,637) were classified as due to reinfection. The proportion of reinfection has remained at around 4% per year (Fig 2). Reinfection was more common among older age groups (>60 years of age, OR = 4.23 *p* = <0.001 vs. those aged <20 years of age) (Table 3). It was also more common among cases with a social risk factor (OR = 1.71, *p* = <0.001), including imprisonment (OR = 1.97, *p* = <0.001), alcohol misuse (OR = 1.66, *p* = <0.001), homelessness (OR = 1.65, *p* = 0.001), and drug misuse (OR = 1.51, *p* = 0.009). TB due to reinfection was also more common among long term migrants (>11 years, OR = 1.80, *p* = <0.001 vs. UK born), and those of black African (OR = 1.40, *p* = <0.001) and white ethnicity (OR = 1.28, *p* = <0.001) compared to those of Indian ethnicity. Reinfection was also more common among migrants from North Africa (OR = 1.84, *p* = 0.037) and sub-Saharan Africa (OR = 1.44, *p* = 0.001) compared to West Europe. The proportion of reinfection among TB cases aged >16 years, by London local authority of residence, 2009 to 2015 were higher for Tower Hamlets (East London, 43/794; 5.4%) and Hackney (North/Central London, 41/561; 7.3%) (S1 Table).

**Table 3:** Univariable analysis of reinfection cases >16 years of age, London, 2009–2015.

	Exposure	Reinfection TB cases			Non- Reinfection TB cases			Odds Ratio	95% confidence interval	p*
		Total	Exposed	%	Total	Exposed	%			
Sex	Male	880	492	55.91	19,220	11,309	58.84	0.89	[0.77–1.02]	0.084
Social risk factors	Any risk factors	766	125	16.32	17,029	1,739	10.21	1.71	[1.40–2.10]	<0.001
	Homelessness	841	46	5.47	18,229	617	3.38	1.65	[1.19–2.25]	0.001
	Prison history	836	40	4.78	18,179	452	2.49	1.97	[1.38–2.75]	<0.001
	Drug use	834	45	5.4	18,140	661	3.64	1.51	[1.08–2.06]	0.009
	Alcohol use	776	54	6.96	17,213	742	4.31	1.66	[1.22–2.21]	<0.001
Site of disease	CNS* TB	880	104	11.82	19,223	2,258	11.75	1.01	[0.81–1.24]	0.948
Time since entry into UK	UK born	803	111	13.82	16,883	2,692	15.95	Reference		
	0–1 years	803	69	8.59	16,883	2,551	15.11	0.66	[0.48–0.89]	0.007
	2–5 years	803	110	13.7	16,883	4,105	24.31	0.65	[0.50–0.85]	0.002
	6–10 years	803	157	19.55	16,883	2,749	16.28	1.39	[1.08–1.78]	0.01
	11+ years	803	356	44.33	16,883	4,786	28.35	1.80	[1.45–2.24]	<0.001
Ethnic group	Indian	879	230	26.17	19,041	5,734	30.11	Reference		
	White	879	108	12.29	19,041	2,097	11.01	1.28	[1.02–1.62]	<0.001
	Black Caribbean	879	19	2.16	19,041	640	3.36	0.74	[0.46–1.19]	0.214
	Black African	879	233	26.51	19,041	4,160	21.85	1.40	[1.16–1.68]	<0.001
	Pakistani	879	89	10.13	19,041	1,899	9.97	1.17	[0.91–1.50]	0.223
	Bangladeshi	879	41	4.66	19,041	1,130	5.93	0.90	[0.64–1.27]	0.561
	Mixed/other	879	159	18.09	19,041	3,381	17.76	1.17	[0.95–1.44]	0.131
World region of birth	West Europe	841	130	15.46	18,496	3,140	16.98	Reference		
	Central Europe	841	28	3.33	18,496	586	3.17	1.15	[0.76–1.75]	0.501
	East Asia	841	8	0.95	18,496	193	1.04	1.00	[0.48–2.07]	0.997
	East Europe	841	9	1.07	18,496	153	0.83	1.42	[0.71–2.85]	0.322
	East Mediterranean	841	8	0.95	18,496	153	0.83	1.26	[0.61–2.63]	0.532
	North Africa	841	14	1.66	18,496	184	0.99	1.84	[1.04–3.25]	0.037
	North America & Oceania	841	0	0	18,496	42	0.23	1.00		
	South Asia	841	348	41.38	18,496	8,769	47.41	0.96	[0.78–1.18]	0.686
	South East Asia	841	36	4.28	18,496	603	3.26	1.44	[0.99–2.11]	0.059
	South, Central America & Caribbean	841	12	1.43	18,496	423	2.29	0.69	[0.38–1.25]	0.217
	Sub-Saharan Africa	841	245	29.13	18,496	4,110	22.22	1.44	[1.16–1.79]	0.001
	Unknown	841	3	0.36	18,496	140	0.76	0.52	[0.16–1.65]	0.265
Age group	16–19	880	16	1.82	19,223	840	4.37	Reference		
	20–29	880	160	18.18	19,223	5,696	29.63	1.47	[0.88–2.48]	0.142
	30–39	880	216	24.55	19,223	5,071	26.38	2.24	[1.34–3.74]	0.002
	40–49	880	166	18.86	19,223	3,052	15.88	2.86	[1.70–4.80]	<0.001
	50–59	880	117	13.30	19,223	1,968	10.24	3.12	[1.84–5.30]	<0.001
	60–69	880	101	11.48	19,223	1,251	6.51	4.24	[2.48–7.23]	<0.001
	70–79	880	72	8.18	19,223	919	4.78	4.11	[2.37–7.13]	<0.001
	80+	880	32	3.64	19,223	426	2.22	3.94	[2.14–7.27]	<0.001

*From Wald test.

### Multivariable analysis of reinfection cases

Factors independently associated with reinfection included having a social risk factor (aOR = 1.96, *p* = <0.001), being a long term migrant (>11 years since entry, aOR = 1.67, *p* = <0.001), and being of black African origin (aOR = 1.46, *p* = 0.001). Reinfection was also more common among female patients and was linearly associated with increasing age (Table 4). Reinfection was also associated with residence in Tower Hamlets (aOR = 2.28, *p* = 0.025) and Hackney (aOR = 2.09, *p* = 0.045) compared to Barking and Dagenham, the area with the lowest proportions (S1 Table).

**Table 4:** Multivariable analysis of risk factors for reinfection, TB patients aged 16 years or older, London, 2009–2015.

Reinfection	aOR**	[95% Conf. Interval]	p>|z|*
0–1 years since entry^1^	0.79	[0.55–1.15]	0.225
2–5 years	0.89	[0.64–1.24]	0.486
6–10 years	1.61	[1.18–2.19]	0.003
11+ years	1.67	[1.25–2.22]	<0.001
Age (linear)	1.02	[1.01–1.02]	<0.001
Sex (male)	0.85	[0.72–0.99]	0.039
Any risk factors	1.96	[1.57–2.45]	<0.001
Black African^2^	1.46	[1.16–1.84]	0.001
Hackney^3^	2.09	[1.02–4.28]	0.045
Tower Hamlets^3^	2.28	[1.11–4.68]	0.025

*From likelihood ratio test

**Adjusted Odds Ratio

^1^UK time of entry versus UK born

^2^Black African versus Indian

^3^Hackney and Tower Hamlets versus Barking and Dagenham

### Multi-drug resistance (MDR) among relapse and reinfection cases

Information on MDR was available for 104 relapse cases from 2009 to 2015. Of these, 19% (20/104) had strains resistant to at least one first line drug: 16% of cases were resistant to isoniazid, and 11% were MDR. Among 454 reinfection cases, 14% (63) were resistant to at least one first line drug: 13% (57) were isoniazid resistant, and 5.1% (23) MDR. Only 18% of MDR TB cases during this time previously had TB (36/200). Most were due to reinfection (23), with 11 due to relapse and the remaining 2 unclassified. In England at this time 8% of incident cases had first line drug resistance, and just 1.7% were MDR [6].

## Discussion

This study has shown a small and decreasing number and proportion of TB cases in London were attributable to relapse. This reduction in relapse cases in London is likely a reflection of improvements in TB control in the UK and elsewhere, including robust treatment pathways and assurance of treatment completion through cohort review and routine surveillance of outcomes. The overall reduction in TB case numbers during this time may also be due to changes in migration patterns (particularly decreasing numbers of migrants from high TB burden countries such as India) [6], as well as a reflection of decreasing TB rates worldwide [8].

We found relapse was associated with a number of factors including being a recent UK migrant (<1 year since entry). These individuals may not have received adequate treatment during their original episode abroad, but we were limited by lack of information on previous treatment that occurred outside of London or prior to 2002, in particular where patients were treated and if the treatment was completed. Site of infection was also an important factor associated with relapse. Our findings show the importance of adequate treatment for patients with CNS, spinal, miliary and disseminated disease to avoid relapse, and support current UK guidance to treat for a minimum of 12 months [9]. Over 10% of relapse patients were infected with MDR strains. Although the original MDR status is unknown, difficult treatment options due to MDR may have been avoidable if drug resistance occurred due to inadequate treatment of the original disease.

Host vulnerability is another key factor associated with relapse particularly among those who may suffer from stress, poverty and deprivation with a history of social risk factors such as homelessness and imprisonment. Other factors such as patients’ immune status may also play an important role in relapse. In addition, social risk factors may be a proxy for patients with previous poor adherence to treatment, increasing their chance of relapse. Strategies to improve treatment adherence among these vulnerable groups are essential to reduce the chance of relapse. This is recognised in recent guidance within the UK NICE guidance for hard to reach groups [10] and is already a priority for the London TB Control Board [11]. Although prevalence of vulnerable groups will vary, these findings are generalizable to services outside of London and the UK.

Reinfection continues to account for around 4% of TB cases per year in London, and reflects repeat or ongoing exposure to TB. Increased risk of exposure to circulating strains may explain the increased level of recurrence seen in London compared to a recent study from another city in England where just 1.8% of patients had a previous history of TB [12]. This study, however, only included those who had repeat episodes of disease in the same city, whilst we included patient reported history including that outside of London and the UK. All four social risk factors including imprisonment, alcohol misuse, homelessness and drug misuse were associated with reinfection, which reflect ongoing exposure within these vulnerable communities. This is likely to be due to exposure to other individuals that may remain undiagnosed or be diagnosed late, and as treatment completion is known to be lower among these groups increasing the risk of being exposed to drug resistant strains. Long-term migrants (>11 years since entry into UK) and individuals of certain ethnicity (black African origin) were also associated with reinfection possibly due to continued risk within established communities. Individuals of older age groups particularly those that are 60 years of age or over have increased risk of reinfection as they have a longer period of life in which they may be re-exposed. In addition, older age is recognised as a risk factor for re-activation, as well as progression following recent exposure. Other factors associated with reinfection included specific geographical areas within London. These boroughs are known to have high rates of TB over this time period, with associated increased risk of exposure to residents of these areas [6].

Furthermore, while many of the factors determining recurrence depend on the patient and their treatment, differences in the *M*. *tuberculosis* genome may have a role in both relapse and reinfection. The introduction of routine whole genome sequencing in PHE (nationally as of January 2018), a highly discriminatory typing tool, will provide useful information on genetic diversity and specific virulence markers between strains [13].

There were a number of limitations in this study including lack of information on HIV status and comorbidities in our surveillance database that may influence recurrence through immunosuppression. Conditions such as HIV infection and diabetes are known to affect both an individual’s risk of developing TB following exposure, and the risk of relapse [14]. This may mean we have not identified the true reasons for relapse or reinfection, and other variables may be a proxy for these underlying causes. Where strain type of both episodes was not available, we had to categorise recurrent TB on other known factors, with a risk of misclassification. We may therefore have incorrectly included relapse cases in the reinfection cohort, and vice versa. This is likely to have weakened the power of our study to detect a true difference, and may be particularly relevant for rarer exposures. Other limitations include incompleteness of sampling, as not all patients were culture confirmed, and only those with isolates from 2010 onwards were strain typed. In addition, there was the possibility of misclassification of strain types due to the use of a less discriminatory molecular typing method (MIRU-VNTR) compared to whole genome sequencing. Furthermore, information on social risk factors was available only from 2009 and details on previous TB diagnosis and treatment was limited where this occurred outside of London (or prior to 2002). We assumed patients with no information on their previous disease had completed treatment. This may mean we included patients who were not fully treated for their previous illness, and were not true cases of relapse or reinfection according to our definition. Although the information collected is routine for all patients, social risk factors may be better elucidated when patients have a previous history of tuberculosis, as clinicians may be more focussed in assessing their needs. This could mean we have overestimated the role of social risk factors in both relapse and reinfection.

It is imperative to share the information we have generated from this study and raise awareness on TB recurrence amongst local TB control services so that they are aware of which patients may be at increased risk of relapse and reinfection. Our findings support current UK guidance on addressing TB in hard to reach groups including active case finding to identify cases earlier and reduce the risk of transmission, and enhanced treatment support, including initiatives such as peer support, addressing housing, mental health and addiction needs, and new initiatives such as video observed therapy to prevent relapse. In addition, the role of contact tracing to identify and prevent further cases remains central to reducing reinfection risk. Monitoring of relapse and reinfection can also be used to assess the effectiveness of TB control programs that are currently in place. This is the first analysis of TB recurrence in London using the national surveillance system and it should inform work of the TB control board and TB services to reduce the burden of recurrent TB. Recurrence due to both relapse and reinfection can be reduced by work with vulnerable and under-served groups to ensure early diagnosis, and strategies to support adequate treatment. There is a current focus on new entrant testing and treatment for latent TB nationally, however this analysis would also support the need for TB awareness raising among those established communities of migrants in London.

## Supporting information

S1 Fig Number and proportion of previously diagnosed TB cases, London, 2002–2015. (DOCX)

S1 Table Multivariable analysis of association between local authority of residence and reinfection cases, 2009–2015. (DOCX)
