# Supplementary material for: Surveillance of tuberculosis (TB) cases attributable to relapse or reinfection in London, 2002-2015
Source: PLoS One. 2019 Feb 15;14(2):e0211972. doi: 10.1371/journal.pone.0211972 (PMC6377187; doi:10.1371/journal.pone.0211972)
Supplement: S1 Fig — (DOCX) [file pone.0211972.s001.docx]

**S1 Fig.** **Number and proportion of previously diagnosed TB cases, London, 2002-2015.**
