# Supplementary material for: Surveillance of tuberculosis (TB) cases attributable to relapse or reinfection in London, 2002-2015
Source: PLoS One. 2019 Feb 15;14(2):e0211972. doi: 10.1371/journal.pone.0211972 (PMC6377187; doi:10.1371/journal.pone.0211972)
Supplement: S1 Table — (DOCX) [file pone.0211972.s002.docx]

**Supporting information**

**S1 Table: Multivariable analysis of association between local authority of residence and reinfection cases, 2009-2015**

| Area | Odds Ratio | P>z | [95% Conf.Interval] |
| --- | --- | --- | --- |
| Barking and Dagenham Reference |  |  |  |
| Barnet | 1.44 | 0.331 | [0.69-3.02] |
| Bexley | 1.00 | 0.999 | [0.34-2.95] |
| Brent | 1.46 | 0.269 | [0.75-2.84] |
| Bromley | 1.67 | 0.399 | [0.51-5.46] |
| Camden | 1.63 | 0.217 | [0.75-3.53] |
| Croydon | 1.22 | 0.6 | [0.58-2.54] |
| Ealing | 1.50 | 0.233 | [0.77-2.93] |
| Enfield | 1.41 | 0.36 | [0.68-2.93] |
| Greenwich | 0.98 | 0.965 | [0.46-2.09] |
| **Hackney** | **2.09** | **0.045** | **[1.02-4.28]** |
| Hammersmith and Fulham | 1.29 | 0.544 | [0.57-2.95] |
| Haringey | 1.87 | 0.077 | [0.93-3.75] |
| Harrow | 1.18 | 0.673 | [0.56-2.49] |
| Havering | 0.89 | 0.838 | [0.28-2.85] |
| Hillingdon | 1.66 | 0.157 | [0.82-3.34] |
| Hounslow | 1.78 | 0.096 | [0.90-3.51] |
| Islington | 1.22 | 0.616 | [0.56-2.69] |
| Kensington and Chelsea | 0.93 | 0.889 | [0.35-2.47] |
| Kingston upon Thames | 1.24 | 0.695 | [0.42-3.67] |
| Lambeth | 1.61 | 0.216 | [0.76-3.44] |
| Lewisham | 1.38 | 0.424 | [0.63-3.04] |
| Merton | 0.72 | 0.5 | [0.27-1.88] |
| Newham | 1.69 | 0.114 | [0.88-3.24] |
| Redbridge | 1.31 | 0.46 | [0.64-2.66] |
| Richmond upon Thames | 2.16 | 0.145 | [0.77-6.07] |
| Southwark | 0.48 | 0.131 | [0.18-1.25] |
| Sutton | 1.39 | 0.492 | [0.54-3.55] |
| **Tower Hamlets** | **2.28** | **0.025** | **[1.11-4.68]** |
| Waltham Forest | 1.59 | 0.205 | [0.78-3.25] |
| Wandsworth | 1.51 | 0.291 | [0.70-3.23] |
| Westminster | 1.20 | 0.659 | [0.53-2.71] |
